# Editorial: At Risk for Neuropsychiatric Disorders: An Affective Neuroscience Approach to Understanding the Spectrum

**DOI:** 10.3389/fnbeh.2016.00165

**Published:** 2016-08-30

**Authors:** Raymond C. K. Chan, Morten L. Kringelbach

**Affiliations:** ^1^Neuropsychology and Applied Cognitive Neuroscience Laboratory, Key Laboratory of Mental Health, Institute of Psychology, Chinese Academy of SciencesBeijing, China; ^2^Department of Psychiatry, University of OxfordOxford, UK; ^3^Center for Music in the Brain (MIB), Aarhus UniversityAarhus, Denmark

**Keywords:** neuropsychiatry, neuroimaging, reward processing, affective neuroscience, pleasure

Neuropsychiatric disorders constitute a large global burden of disease; yet the neural mechanisms of emotional and social cognitive disturbances in patients are not yet fully understood (Deco and Kringelbach, 2014). Here, we used an affective neuroscience approach to investigate the spectrum of neuropsychiatric disorders from patients to those at risk, where stress plays a major role (Sousa, 2016). This Research Topic illuminates work by leading affective neuroscience researchers on how best to understand problems with the affective processing leading to increased risk for neuropsychiatric disorders.

The 20 articles in the Research Topic can roughly be divided into five sections: 1. Overviews, 2. Methods, 3. Anxiety and Personality, 4. Social Cognition, and 5. Impulsivity/Compulsivity, although many papers will fit in multiple categories. In the following we briefly introduce each article in this context.

## Overviews

Anhedonia is an underlying critical feature of neuropsychiatric disorder but has remained little understood since first being introduced by Theodore Ribot in 1896 as the “inability to experience pleasure” (Ribot, 1896). Rømer Thomsen and colleagues proposes to reconceptualize anhedonia as an impairment to the established pleasure mechanisms of wanting, liking and learning (Rømer Thomsen et al.).

Cognition interacts with emotion to play a major role in those at risk for developing neuropsychiatric disorders. In particular Petrovic and Castellanos identified the importance of the interaction between deficient cognitive top-down executive control and emotional dysregulation in neuropsychiatric patients. They develop a novel framework that can integrate attention-deficit/hyperactivity disorder (ADHD), emotional traits in ADHD, borderline and antisocial personality disorder into a related cluster of mental conditions.

Still, some neuropsychiatric disorders are not fully understood. Sallin et al. describe investigations into resignation syndrome, which is long-standing disorder predominately affecting psychologically traumatized children and adolescents in the midst of a strenuous and lengthy migration process. They evaluate several hypotheses including stress and culture-bound psychogenic catatonia, and show how models of predictive coding and in particular negative expectations could explain the down-regulation of higher order and lower order behavioral systems in vulnerable individuals.

Genetic factors are also important for the development of neuropsychiatric disorders. Xia and Yao conducted a systematic review of the genes involved in adolescent depression and found positive association between *BDNF* Val66Met genotype and adolescent depressive symptoms (Xia and Yao).

## Methods

Animal models can potentially provide new insights into the neural mechanisms of neuropsychiatric disorders. Durieux and colleagues tested the hypothesis that the known imbalance between excitatory glutamate and inhibitory GABA transmission found in some neuropsychiatric disorders can be indirectly modulated through glial mechanisms (Durieux et al.). They used mice to test that striatal glutamate levels can be shifted by N-acetylcysteine and found that the treated animals were significantly less active and more anxious in the open field test.

Still, studies on human participants are likely to be more informative and objective measurements of relevant impairments are urgently needed. In order to address this, Bland and colleagues developed a standardized test battery, comprised of existing, adapted and novel tasks, to assess four core domains of affective cognition (emotion processing, motivation, impulsivity and social cognition) in order to facilitate and enhance treatment development and evaluation in a broad range of neuropsychiatric disorders (Bland et al.).

The treatment of neuropsychiatric disorder has a long history but the development of deep brain stimulation (DBS) for the treatment of Parkinson's disease is relatively recent (Kringelbach et al., 2007). The therapeutic effects are thought to arise from a rebalancing of brain-wide networks (Kringelbach et al.) but only recent developments in methods including probabilistic tractography and whole-brain computational modeling enabled Van Hartevelt and colleagues to identify the Hebbian-like changes in structural connectivity induced by long-term deep brain stimulation (van Hartevelt et al.).

## Anxiety and related disorders

Anxiety is a significant neuropsychiatric problem, especially in adolescence. Geng and colleagues investigated adolescents' susceptibility to trait anxiety (Geng et al.) and found that weaker intra- and inter-network functional connectivity of the salience network was linked to higher trait anxiety among adolescents, which may underlie altered salience processing and cognitive regulation.

Similarly, Gawda and Szepeitowska investigated the brain activity in low/high-anxious adult groups of participants based on measures of trait anxiety (Gawda and Szepietowska). They found significant differences during the performance of more complex tasks between individuals with low anxiety and those with high anxiety.

Adolescents are also prone to develop compulsive behaviors which include various forms of behavioral addictions (Blakemore and Robbins, 2012). Wang and colleagues studied the structural brains of adolescents with internet gaming disorder and found it is linked to significant alterations of gray matter volume associated with performance change in cognitive control (Wang et al.).

Borderline personality disorder is another important neuropsychiatric disorder linked with differences in brain activity. Using EEG, Van Elst and colleagues found significant intermittent rhythmic delta and theta activity in patients compared to controls (Tebartz van Elst et al).

## Social cognition

Depression has been strongly linked to social functioning (Stroud et al., 2011). Dedovic and colleagues used neuroimaging to show that the dorsal anterior cingulate cortex responds to repeated bouts of negative personally-relevant social evaluations (Dedovic et al.). This reveals a new dimension to this brain region in processing exclusion and contributing to mental health outcomes in those vulnerable to depression.

In more evidence of how depression is linked to impaired social functioning, Young and colleagues found that depression reduces psychomotor performance and alters affective movements in caregiving interactions based on salient affective sounds (Young et al.).

More evidence of changes in social cognition was unearthed in a neuroimaging study of voice perception and handedness which showed that a polymorphism specifically affects voice-specific brain function (Koeda et al.).

Finally, Wang and colleagues used neuroimaging to examine participants with different risk for psychosis (Wang et al.). They found that different dimensions of schizotypy are correlated with brain regions involved in social cognitive abilities.

## Impulsivity/Compulsivity

The naturally occurring behaviors of impulsivity and compulsivity can become pathological and as such are key examples of neurocognitive endophenotypes with possible cross-diagnostic significance, linked to co-morbidities and commonalities across a range of neuropsychiatic disorders (Robbins et al., 2012).

Problematic hypersexual behavior is a compulsive behavioral compulsive disorder defined as the continuous participation in sex acts with little or no control over excessive sexual compulsivity and behavior despite the awareness of the associated negative outcomes. Seok and Sohn used neuroimaging to study a group of hypersexual individuals and found significant differences compared to controls, which are consistent with other behavioral addictions (Seok and Sohn).

Hoarding disorder is another compulsive disorder, characterized by the excessive acquisition and retention of goods with limited or no value, leading to significantly cluttered living spaces, and significant associated distress and life impairment. Vickers and colleagues used behavioral methods to investigate hoarding disorder and found that individuals in the high hoarding group were more impatient for consumables than for monetary reward, which suggest a specific, potent desire for consumable rewards (Vickers et al.).

Tahmasian and colleagues used positron emission tomography to investigate correlations between glucose metabolism and impulsivity in patients with Parkinson's disease (Tahmasian et al.). They found that high impulsivity is associated with increased metabolism within the fronto-insular network.

The anticipation of reward is an important component of the pleasure system which can become unbalanced in neuropsychiatric disorders, especially in adolescence. Li and colleagues used neuroimaging to investigate anticipatory pleasure in healthy adolescents and found that activity in the mesolimbic pathway during the anticipation of monetary reward could to some extent be predicted by subjective anticipatory pleasure (Li et al.).

Anticipation of reward has been studied in the context of delayed discounting and intertemporal decision-making. Ludwig and colleagues used neuroimaging to uncover the neural correlates of delay discounting during reward delivery and found that impulsivity and subclinical anxious-depressive traits are related to stronger neural responses for winning immediate relative to delayed rewards (Ludwig et al.).

## Conclusion

Overall, this collection of 20 works of original research and reviews provides both novel ideas and a novel foundation upon which to build future research into the functional neuroanatomy of the spectrum of neuropsychiatric disorders. Drawing upon a wide variety of fields and using a diverse set of methods, techniques, and populations, this body of work points to some exciting future avenues of research.

## Author contributions

All authors listed, have made substantial, direct and intellectual contribution to the work, and approved it for publication.

## Funding

RC is supported by a grant from the “Strategic Priority Research Program (B)” of the Chinese Academy of Sciences (XDB02030002), the National Science Fund China (81088001, 91132701), the Beijing Training Project for Leading Talents in S&T (Z151100000315020). MK is supported by an ERC Consolidator Grant CAREGIVING (n.615539).

### Conflict of interest statement

The authors declare that the research was conducted in the absence of any commercial or financial relationships that could be construed as a potential conflict of interest.
